# A systematic review of media parenting in the context of childhood obesity research

**DOI:** 10.1186/s12889-016-2981-5

**Published:** 2016-04-14

**Authors:** Alyssa Aftosmes-Tobio, Claudia Ganter, Selma Gicevic, Sami Newlan, Christine L. Simon, Kirsten K. Davison, Jennifer A. Manganello

**Affiliations:** Department of Nutrition, Harvard T.H. Chan School of Public Health, 665 Huntington Avenue, Boston, MA 02121 USA; Department of Epidemiology, Harvard T.H. Chan School of Public Health, Boston, Massachusetts USA; Department of Social and Behavioral Sciences, Harvard T.H. Chan School of Public Health, Boston, Massachusetts USA; Department of Health Policy, Management and Behavior, University at Albany, SUNY, Albany, New York USA

**Keywords:** Childhood obesity, Media parenting, Home media environment, Systematic review

## Abstract

**Background:**

We conducted a systematic review to obtain studies on childhood obesity and parenting published between 2009 and 2015, and draw out those studies with a particular focus on media parenting. Our analysis addresses two major aims: 1) to describe how media use and media-related parenting practices and skills are operationalized in studies and 2) to explore whether studies measured ecological factors (e.g. individual-, family-, and community-level factors), which could be associated with media parenting practices.

**Methods:**

Using a standardized, multi-stage process, we identified and screened articles focused on parenting and childhood obesity (*N* = 667). Studies were eligible for this analysis if they measured media parenting and/or the home media environment, resulting in a sample of 103 studies. We used quantitative content analysis to code the full text articles for content related to our study aims; analyses were performed using SAS 9.4.

**Results:**

Seventy nine percent of studies measured media use, 82 % measured media parenting, and 65 % measured the home media environment. Studies measuring media use focused on a limited number of devices; while all studies measured child/parent use of televisions, only 3 % measured use of smartphones, 1 % measured use of laptops, and no studies measured use of tablets. Measures of parenting practices focused largely on rules specific to limiting screen time. Although 60 % of studies measured at least one ecological factor, child-specific and neighborhood/community-level factors were rarely measured.

**Conclusions:**

More detailed measurements of media use that reflects current technology trends and diverse contexts of use are needed to better understand media use and parent regulation of child media exposure. Measures of the ecological context can more fully assess factors impacting media parenting and, subsequently, child risk for overweight and obesity.

**Electronic supplementary material:**

The online version of this article (doi:10.1186/s12889-016-2981-5) contains supplementary material, which is available to authorized users.

## Background

The American Academy of Pediatrics (AAP) recommends children spend no more than 1 to 2 h per day using screens [1], yet, it has been documented that children spend more time using screen media than attending school [2]. Screen media includes televisions (TV), video gaming systems, computers, cell- or smartphones, and other electronic devices such as tablets or laptops [3, 4]. According to a recent Kaiser Family Foundation report, children ages 8 to 10 years-old spend nearly 8 h using media each day, while teenagers spend more than 11 h [5]. While there is an evidence base on the harmful effects of TV viewing on children’s risk of obesity [6–8], we know much less about the effect of modern screen based devices such as smartphones and tablets [9]. Rapidly evolving forms of media demand creativity and adaptability on the part of researchers looking to capture device use among children and make recommendations to parents on how to intervene on their child’s use.

**Fig. 1:**
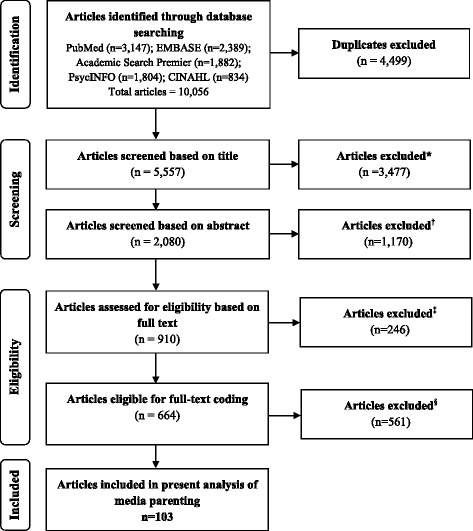
PRISMA flowchart: summary of process to identify and screen eligible articles on media parenting and childhood obesity published between 2009 and 2015. Footnote: *Exclusion criteria: unrelated topic; children not focus of the study; not written in English; animal study; focuses on specific medical population; duplicate; manually deleted duplicates; ^†^Exclusion criteria: unrelated topic(*n* = 210); intervention (*n* = 430); no parent research participants (*n* = 218); not focused on parenting (*n* = 261); not relevant to childhood obesity (*n* = 45); duplicate (*n* = 6); ^‡^Exclusion criteria: missing PDF, duplicate, dissertation, retraction, or conference abstract (*n* = 49); not research (*n* = 9); not written in English (*n* = 4); parents not research participants (*n* = 43); intervention study (*n* = 29); not focused on parenting (*n* = 58); not relevant to childhood obesity (*n* = 21); articles from the same study sample (*n* = 33); ^§^ Exclusion criteria: no measure of media parenting and/or the home media environment

Parents shape child behaviors through modeling, guiding their children through reinforcement, and controlling their environments [10–14]. They play a significant role in monitoring child media consumption and helping them find alternative activities. Although studies have previously explored parenting practices associated with child exposure to specific types of media content, there has been limited research focused on the implications of media parenting practices for obesity prevention [3]. Jago, et al. recently published a systematic review [13] of 29 studies examining associations between media parenting and child screen viewing, with a particular focus on parenting measures. The authors reported inconsistent relationships between parenting and child screen use, and highlighted important shortcomings in parenting measures. This review did not focus on childhood obesity, nor did it provide a systematic and quantitative assessment of how child media use was operationalized.

As a result of the dramatic proliferation in child screen use, there is an increasing need for interventions to support parent modulation of child screen use. It is questionable, however, if the literature is at a stage of development to support the creation of such programs. Thus, at this time it is important to take stock of the available evidence. In particular, it is important to document the specific media devices and media parenting practices measured in studies, and the extent to which studies have examined the ecological context. This information is crucial to understand gaps in the literature that must be addressed. With this in mind, we conducted a systematic review to identify studies on childhood obesity and parenting, and draw out those studies with a particular focus on media parenting. By doing this, our analysis addresses two major aims: 1) to describe how media use and media-related parenting practices or skills are operationalized in studies; and 2) to explore whether studies included measures of ecological factors, which could be associated with media parenting.

## Methods

For this study, a multi-stage process was used to: 1) identify articles on parenting in the context of childhood obesity; 2) screen for eligibility; and 3) compile a final sample of studies for full-text coding. Five databases (PubMed, EMBASE, Academic Search Premier, PsycINFO, and CINAHL) were searched to identify studies for inclusion. The final database search was conducted in December 2015 (see Fig. 1). We used search terms related to parents or parenting, and obesity or obesity-related risk behaviors (see Additional file 1 for an example of search terms used); included search terms related to media use, devices, and media parenting; and limited our search to parents/caregivers of children 0–18 years of age. We also used terms to exclude unrelated topics unrelated, such as family history or pregnancy, as well as non-research studies (e.g., letters, reviews, commentaries). A total of 5557 unique studies were identified after removing 4499 duplicates. An initial screening of study titles eliminated those meeting the following exclusion criteria: focused on unrelated topics (e.g. food safety), were not written in English, included animals, targeted a clinical population (e.g. children with spina bifida), or were not original research (e.g. commentaries, letters), resulting in a pool of 2080 studies. Two coders then applied eligibility criteria to study abstracts. Eligible studies included non-intervention, peer-reviewed research studies written in English that focused on parenting (e.g. measured parenting behaviors, styles, or cognitions), were relevant to childhood obesity (e.g. diet, sedentary behavior), included parents/caregivers as research participants, and were published between January 2009 and December 2015. This time parameter was chosen to capture the most recent literature available and for the feasibility of coding, given the high volume of studies identified in this seven-year time period alone. Intervention studies were excluded from the sample because they require a separate coding scheme, which was beyond the scope of the present study. Consistent with systematic review guidelines [15] only one article per study was included in the analysis. When multiple articles from the same study were identified, we included the first published article. When multiple articles from the same study were first published in the same month, we randomly selected one article to include in the sample.

The two coders achieved 90 % agreement in applying the eligibility criteria, and all discrepancies were discussed and resolved. A total of 1170 studies did not meet the eligibility criteria or could not be located, resulting in a sample of 667 studies representative of recent literature on parenting and childhood obesity. A separate analysis was conducted on the total pool of eligible studies and is reported elsewhere [16]. Studies were eligible for the present analysis if they were coded as measuring media parenting practices (e.g., rules around TV/computer/video game or other media use) and/or the home media environment (e.g., parents’ own media use, the presence of a media device the bedroom), resulting in a final sample of 103 studies.

### Coding procedures

We extracted data from eligible studies using quantitative content analysis, which is the systematic assessment of content using categorical rules, and use of statistical methods to describe patterns in the content analyzed [17]. This method has been used in other studies of academic literature [18–20]. We developed a comprehensive codebook to standardize all coding procedures, and the 103 eligible studies were reviewed and coded by two trained coders.

Study characteristics coded include year of publication, geographic region, target child age, and study methodology type (e.g. quantitative, longitudinal). Parent/caregiver participant categories include sex, underserved groups (e.g. low-income/socioeconomic status [SES]), and ethnic/racial categories. Child and/or parent media use were coded if measured. Where media use was measured, the following were subsequently coded: data collection tool (e.g., questionnaire, log/journal); respondent reporting media use (child/parent); devices measured (e.g., TV, computer, smartphone); and other modes of child media exposure (e.g., media in the child’s bedroom, screen use during mealtime). For media parenting, media-related parenting practices (e.g., media rules, parent-child co-viewing, parent use of screen time for behavioral management) were coded, along with parent skills related to media use (e.g. media literacy, self-efficacy to control screen use). Finally, using the Family Ecological Model [21], we created categories for measures of the family ecology and social/emotional context factors including individual (e.g. child attributes), familial (e.g. parent cognitions), and community-level (e.g. neighborhood) factors. The Family Ecological Model conceptualizes the complex and nuanced environments within which families operate [21], and provides a comprehensive framework available for examining the environmental factors associated with parenting around child obesogenic behaviors.

### Inter-coder reliability and analysis

We assessed inter-coder reliability using both simple percent agreement and the Krippendorff’s alpha (k-alpha; α) coefficient [22] for each coding category by double-coding a randomly selected sample of eligible studies (15 %; *n* = 15). We calculated reliability statistics across all 15 studies using STATA 13, resulting in an average k-alpha of 0.83 (range: 0.6–1.0), and average simple percent agreement of 0.95 (range 0.8–1.0). All variables fell into an acceptable range of reliability. Twelve variables remained in our analysis although the k-alpha statistic was 0.0 because the simple agreement was 90 %; α =0 indicates a lack of variability in the ratings of each variable, which occurred because those variables were rarely identified. We based our decisions for variable inclusion and cutoff points on previously documented recommendations [22–24].

Variable frequencies were calculated by variable using SAS 9.4 (Cary, NC), and each table presents the number of studies that include the specified characteristic, and the percentage of all eligible studies.

## Results

Of the 103 eligible studies identified, media parenting (*n* = 36), the home media environment (*n* = 22), or both (*n* = 45) were measured (see Table 1). Studies were spread fairly evenly across the years of publication, although fewer studies were identified in 2009. Over half of studies originated in the United States (U.S.) (53 %) and a little over a quarter from Europe/United Kingdom (27 %). Target children were mostly preschool (38 %), elementary school (52 %), and middle school (43 %) ages; infants/toddlers were included in only 5 % of studies and high school aged children in 13 % of studies. Studies predominantly utilized quantitative methods (97 %) and adopted a cross sectional design (89 %). Although most studies included both male and female parent/caregiver participants (55 %), sample size distribution between males and females was quite uneven. Eighty eight percent of studies that included both male and female participants had female sample sizes that were *n* = 101 or more (versus 35 % of male samples), but 58 % of male participant sample sizes were *n* = 100 or less (versus 12 % of female samples). A quarter of studies did not specify the sex of the participating parents. Although over a third of studies targeted racial/ethnic minority parents during recruitment, only 18 % of studies included Asian parents/caregivers and 11 % included Indigenous parents – these numbers remained constant for U.S.-based studies as well. Fewer than one in five studies targeted parents from low-SES backgrounds, and less than one in ten targeted immigrant parents.

**Table 1:** Study and parent/caregiver characteristics (*N* = 103)

Study characteristic	Number	Percent
Year		
2009	5	5 %
2010	13	13 %
2011	17	16 %
2012	14	14 %
2013	15	15 %
2014	23	22 %
2015	16	15 %
Geographic region		
United States	55	53 %
Europe/United Kingdom	28	27 %
Australia/New Zealand/Papua New Guinea	10	10 %
Asia	6	6 %
Canada	3	3 %
Mexico/Central America	1	1 %
Age ranges^a^		
0–1 years (infant/toddler)	5	5 %
2–5 years (preschool)	39	38 %
6–10 years (elementary school)	54	52 %
11–13 years (middle school)	44	43 %
14–18 years (high school)	13	13 %
Study Methodology^a^		
Quantitative methods (vs. qualitative or mixed methods)	100	97 %
Longitudinal (vs. cross sectional)	11	11 %
Parent/Caregiver Characteristics	N	%
Sex of parent participants		
Both males and females	57	55 %
Females only	20	19 %
Males only	0	0 %
Not specified	26	25 %
Underserved groups targeted in recruitment^a^		
Racial/ethnic minority parents	36	35 %
Low income/education/socioeconomic parents^b^	19	18 %
Immigrant parents	9	9 %
Ethnic/Racial groups included^a^		
White, Non-Hispanic	44	43 %
Black/African American	37	36 %
Hispanic	35	34 %
Asian	19	18 %
Indigenous	11	11 %
Media-specific Measurements^a^	N	%
Media parenting	84	82 %
Media use	81	79 %
Home media environment	67	65 %

^**a**^More than one answer could be selected, therefore totals may not equal 100 %; ^b^Income or education; includes recipients of income-eligible Federal assistance programs

A total of 81 studies - 79 % of our sample - measured media use (see Table 2). Studies reported media use of children (36 %), parents (7 %), or both (36 %), and reporting of use corresponded with child age; media use was reported by parents for the vast majority of studies with children ages 0–5 (97 %), while both parent- and child-reported measures were used for the majority of studies with children ages 14–17 (75 %). Studies relying on child-only reporting were limited to children ages 6–17. The vast majority of studies measured media use using questionnaires (98 %). Less than half of studies (43 %) provided information on the specific devices used; the remaining studies collapsed such information across multiple devices, making it impossible to parse the results for each device. For example, while TV was included in all studies measuring device use, it was measured distinctly from all other devices in only 43 % of the studies. One in five studies reported the use of video games or computers separate from other devices, and report of use specific to smartphones or laptops was rare (1–3 % of studies). No studies measured tablet use. Over a third of studies measured the presence of media in the child’s bedroom, most often the presence of a TV (34 %); however, as was the case for media use, measurement of the presence of newer technologies - laptops, tablets, or smartphones - in a child’s bedroom was rare (5 %). Less frequently measured modes of child media exposure include screen use during mealtime (17 %), the number of devices in the home (17 %), and passive media exposure (e.g. finding the TV turned on when arriving home from school) (4 %). Measures of media use were also limited to the home environment; only one study measured media use outside the home, in this case in a child care setting.

**Table 2:** Measures of media use (*N* = 103)

Characteristic	Number	Percent
Media use measured		
No	22	21 %
Yes^a^	81	79 %
Child use only	37	36 %
Parent use only	7	7 %
Both parent and child use	37	36 %
Method used to measure media use		
Questionnaire	79	77 %
Log/journal	1	1 %
Video diary	0	0 %
Other	1	1 %
Respondent reporting media use		
Parent reported	55	53 %
Child reported	5	5 %
Both parent and child reported	20	19 %
Devices Measured^a^		
Device-specific data are available	44	43 %
TV	44	43 %
Video games	21	20 %
Computer	21	20 %
DVD/video player	12	12 %
Smartphone	3	3 %
Laptop	1	1 %
Tablet	0	0 %
Device-specific data are not available^b^	37	36 %
Other measures of child media exposure^a^		
Media in the child’s bedroom	38	37 %
TV	35	34 %
Computer	16	16 %
Video games	11	11 %
DVD/video player	7	7 %
Laptop	3	3 %
Tablet	1	1 %
Smartphone	1	1 %
Screen use during mealtime	18	17 %
Number of devices in home	17	17 %
Passive media exposure	4	4 %

^a^More than one answer could be selected, therefore totals may not equal 100 %; ^b^Multiple devices were measured in a single question (e.g., time per day watching TV/playing video games) resulting in no device-specific information

Media parenting practices were assessed in 82 % of studies (see Table 3). The most common media parenting construct measured in studies related to rules around media use (57 %), and the majority of those studies focused on time limits. The study of rules specific to media use during meals (4 %), media in children’s bedrooms (3 %), media use at specific times of the day (3 %), media use in relation to other activities (6 %), and on media use weekdays versus weekend days (1 %) was much less common. Following media rules, parent modeling of screen use, including parent-child co-viewing, was the next most commonly measured media parenting construct (42 %). Far fewer studies measured parents’ use of screen time for behavior management (11 %), or parent encouragement of screen use (2 %). Measurement of parenting skills around media use was also low; parental beliefs, attitudes, and intentions were measured in 14 % of studies and parents’ media-specific self-efficacy in only 16 %. Results showed that 94 % of studies did not include any measure of parent media literacy.

**Table 3:** Media parenting and family context constructs measured in eligible studies (*N* = 103)

Characteristic	Number	Percent
Parenting Practices		
Media rules^a^	59	57 %
Time limits	46	45 %
Screen use in relation to other activities	6	6 %
Screen use during meals	4	4 %
Time of day screens can be used	3	3 %
Media use in child’s bedroom	3	3 %
Weekday vs. weekend screen use	1	1 %
Other	17	17 %
Parent modeling of screen use (including parent-child co-viewing)	43	42 %
Parent use of screen time for behavior management	11	11 %
Parent encouragement of screen use	2	2 %
Parenting Skills		
Parent beliefs/attitudes/intentions about media use	14	14 %
Parental self-efficacy related to child screen use	16	16 %
Parent media literacy	6	6 %
Ecological context factors^a^	62	60 %
Intrafamilial dynamics	36	35 %
Parent cognitions	30	29 %
Neighborhood/community	13	13 %
Child attributes	6	6 %

^a^More than one answer could be selected, therefore totals may not equal 100 %

Forty three studies (42 %) measured a single ecological factor; 17 % measured multiple factors. Family-level factors include parent cognitions (i.e. knowledge, beliefs, attitudes, intentions) and intrafamilial dynamics and were measured in about a third of studies (29 and 35 %); however, contextual factors at other levels were not often measured. Since child media use, exposure, and parenting is centered at home, we wanted to explore measurement of additional elements within the intrafamilial domain which could affect parenting practices and skills [25], such as parents’ ability to set rules around screen time. Four percent or less of studies measured any of these factors, including parental self-efficacy (4 %), mental health (4 %), social support (2 %), parent stress (2 %), social norms (1 %), parental control (1 %), food insecurity (1 %), acculturation (1 %), and child care challenges (1 %). No studies measures housing instability, family conflict, or parent self-esteem.

## Discussion

Given the ever-increasing ubiquity of media and screens in children’s lives [5, 17] and the documented relationship between screen use and childhood obesity [26–28], the paucity of studies measuring media use and media parenting within the literature of parenting and childhood obesity is concerning. Results from this analysis can provide explicit guidance on future work needed in this area.

The vast majority of studies on media parenting originated from the United States, Europe/United Kingdom, and Australia. Although child obesity rates are increasing in low/middle income regions [29], there is a lack of representation of those areas in our sample, with only 7 % of studies originating from Asia and Mexico/Central America, and no studies from Africa or South America. While it is possible that there are still disparate levels of screen use in low/middle versus high-income countries, which would temper the association between screen behaviors and childhood obesity, we cannot know this without measurement.

Our analysis examined parent populations targeted for participation, and found more than half of studies included both female and male parent participants, which presents an opportunity to engage both mothers and fathers around media parenting as part of efforts to reduce child obesity. However, greater work is clearly needed in this area, as we found that sample size distributions between male and female participants were skewed to include many more mothers/female caregivers than fathers/male caregivers. These disparities are addressed in a forthcoming manuscript. Our study also revealed other shortcomings in specific parent group inclusion. Although parenting around obesogenic behaviors remains important in adolescence [28, 30], parents of teenagers were also largely absent from the studies we reviewed.

While 35 % studies included minority parents, the lack of inclusion of Asian and Indigenous parents, particularly in U.S.-based studies, is concerning given that Asians are the fastest growing minority group in the U.S., followed by Native Hawaiian/Pacific Islanders [31]. It has been suggested that parent attitudes and beliefs about media use vary by culture [32], and that youth media use varies by race/ethnicity [33–36], so it is important to include parents across multiple racial/ethnic groups to document those nuances. It is concerning that a limited number of studies recruited low income/SES parents, given the pervasiveness of childhood obesity and the fact that barriers experienced by parents in these groups have been documented [37, 38]. Specific recommendations for recruiting under-resourced groups have been previously noted [39, 40]; we would add that our analysis highlights the continued need for protocols that specifically target low-income parents in order to document persisting disparities, identify the circumstances within which people are parenting, and to strengthen strategies to target underserved populations.

Our analysis shows that measures of child media use in studies focused on childhood obesity are largely centered on television viewing, which does not reflect the varied modes of exposure children experience. This could potentially be explained by low popularity of “newer’ devices (e.g. tablets) among children, particularly in the earlier publication years included in our analysis. However, reports about the changing landscape of child device use [41], and rapid increases in such use [42] indicate that child use of these devices was already underway in the earlier years of our search parameters. Additionally, while several studies measured the presence of such devices in children’s bedrooms (i.e. laptops, smartphones, and tablets), only one study measured use of laptops, and no studies measured parent or child use of tablets. We encourage researchers to focus on capturing the full landscape of media devices used by children in future research. Many studies measuring media use utilize questions that collapse device categorization (e.g. defining computers as “desktops/laptops/tablets”), creating unspecified results which obscures device-specific media use by children. As some devices lend themselves to potentially increasing child exposure (e.g. portable devices such as handheld gaming devices versus stationary devices such as TVs), accurately documenting child media exposure by device-type is crucial to understanding their exposure across time and settings, as well as how parents are or are not setting bounds to that exposure.

Relatedly, our analysis shows that although data on media parenting is being collected, measures generally focus on the existence of rules regarding time limits and do not account for settings outside of the home, where children could be using media, such as in the car on the way to school or during afterschool programs. Additionally, greater consideration should be given to measuring the ways media is used as a mode of parenting, such as the use of media for managing child behavior, incidence of media viewing as a family activity, or screen use during meals. More work is needed in order to better understand the ways in which children are using media and how parents are allowing or responding to that use.

Another surprising omission from this literature is measurement of parent media literacy, or other relevant skills such as digital literacy. Measures of media literacy in the literature typically focus on assessments among children or parent acquisition of media literacy skills [43, 44]. Consistent definitions and validated measures of this are currently lacking, but are needed in order to understand and quantify existing parent knowledge about media. Parents cannot provide proper supervision of media they are not familiar with.

Observational studies on parenting and childhood obesity, particularly as precursors to intervention efforts, are meant to have a thorough understanding of parent experience, which should include accounting for the contexts within which parenting occurs. Family ecology and social and emotional contexts shape and drive families’ day-to-day experiences and behaviors. Our analysis shows that more attention could be paid to neighborhood/community factors, including social capital, neighborhood safety, and deprivation. Child-specific factors, such as temperament and self-regulation were also absent from the literature. Just as with family-specific factors, these domains may also impact parenting practices around media use, and their measurement can inform behavior change strategies or associations with risk of child overweight.

This analysis is not without limitations. We restricted our review to English-language and non-intervention studies, which may have limited our final sample of studies available for analysis. We refined our initial search terms to collect studies that included parents as research participants and those with a focus on parenting related to childhood obesity (i.e. parenting practices related to diet, physical activity, and sedentary behaviors). In so doing, we will not have captured studies that explore parenting solely related to media content, and may have missed, for example, studies that measure parent regulation of television food advertisements and commercials. By placing these limits on our search terms, we avoided having many irrelevant studies in our initial sample of studies, and we are confident that our sample accurately reflects the literature on parenting of media specifically as it relates to childhood obesity. An area of future research could be an analysis of measurements of the content or platforms of media used by children, such as active gaming or social media. We did not assess risk of bias across studies or strength of evidence for study outcomes because these were not relevant to our research aims. There are also many strengths of this analysis. The studies reviewed were drawn from a broad range of publication disciplines, included both U.S.-based and non-U.S. studies, broadly examined media parenting in the context of childhood obesity, and examined the presence of environmental factors that may impact screen viewing and parenting practices thereof.

## Conclusions

More detailed measurements of media use that reflects current technology trends and diverse contexts of use are needed to better understand media use and parent regulation of child media exposure. Future observational studies should consider recruitment protocols which target more diverse samples of parents, and consider including measures of the ecological context within which families live to more fully understand factors that may impact media parenting and, subsequently, child risk for overweight.

### Ethics approval and consent to participate

Not applicable.

### Consent for publication

Not applicable.

### Availability of data and materials

The dataset supporting the conclusions of this article are available upon request to the corresponding author.

## Additional files

Additional file 1: Search Terms (PubMed). (DOCX 14 kb)

## Abbreviations

SES: socioeconomic status
TV: television
U.S.: United States
