# Supplementary material for: A systematic review of media parenting in the context of childhood obesity research
Source: BMC Public Health. 2016 Apr 14;16:320. doi: 10.1186/s12889-016-2981-5 (PMC4831097; doi:10.1186/s12889-016-2981-5)
Supplement: Additional file 1: — Search Terms (PubMed). (DOCX 14 kb) [file 12889_2016_2981_MOESM1_ESM.docx]

Additional File: Search Terms (PubMed)

Search term [search condition]

(“Parent-Child Relations” [MeSH Terms] OR parenting[tw] OR parenting styles[tw] OR feeding style[tw] OR feeding styles[tw] OR parenting practice[tw] OR parenting practices[tw] OR parental influence[tw] OR parental influences[tw] OR family[ti] Or familial[ti] OR families[ti] OR mother[ti] OR mothers[ti] OR mother’s[ti] OR father[ti] OR fathers[ti] OR father’s[ti] OR parental encouragement[tw] OR parental support[tw] OR parental provision[tw] OR parental factors[tw] OR parenting efficacy[tw] OR parental monitoring[tw] OR parental modeling[tw] OR parental modelling[tw] OR home influence[tw] OR home food availability[tw] OR parent mediation[tw] OR rule making[tw] OR mealtime*[tw] PR parent restriction*[tw] OR parental restriction*[tw] OR [primary caregiver[ti] OR primary caregivers[ti] OR caregiving practice*[tw])

AND

(obesity[tw] OR body weight[tw] OR overweight[tw] OR weight status[tw] OR body mass index[tw] OR adiposity[tw] OR obesogenic[tw] OR portion size[tw] OR physical activity[tw] OR sedentary[tw] OR exercise[tw] OR screen time[tw] OR television[tw] OR TV[tw] OR computer[tw] OR video game[tw] OR video games[tw] OR family meals[tw] OR active play[tw] OR outdoor play[tw] OR food intake[tw] OR eating behavior[tw] OR eating behaviors[tw] OR eating behaviours[tw] OR eating behaviours[tw] OR snack[tw] OR snacks[tw] OR snacking[tw] OR food rewards[tw] OR food reinforcement[tw] OR BMI[tw] OR fruit[ti] OR vegetable[ti] OR vegetables[ti] OR feeding patterns[tw] OR food consumption[tw] OR dietary behavior*[tw] OR dietary behavior*[tw] OR sweetened beverage*[tw] OR food choice*[tw] OR media monitoring[tw] OR media exposure[tw] OR media use[tw])

NOT

(comment[ptyp] OR editorial[ptyp] OR letter[ptyp] OR case reports[ptyp] OR news[ptyp] OR review[ptyp] OR family adjustment[tw] OR family history[tw] OR family resemblance[tw] OR family planning [tw] OR family structure[tw] OR family income[tw] OR rat[tw] OR rats[tw] OR prenatal[tw] OR preconception*[tw] OR pregnancy[tw] OR pregnancies[tw] OR pregnant[tw] OR malnutrition[tw] OR malnourished[tw] OR breastfeeding[tw] OR breast-feeding[tw] OR perinatal[tw] or postpartum[tw] or neonatal[tw])
